# Evaluation of Myocardial Blood Flow Using ^18^F-flurpiridaz PET: Established Applications with Pharmacologic Stress and Roadmap for Exercise Stress Implementation

**DOI:** 10.1007/s11886-026-02439-4

**Published:** 2026-10-23

**Authors:** Farzana Z. Ali, Piotr J. Slomka, René R. Sevag Packard

**Affiliations:** 1 Ahmanson Translational Theranostics Division, Department of Nuclear Medicine and Theranostics, University of California Los Angeles (UCLA), Los Angeles, CA USA; 2 Departments of Medicine (Division of Artificial Intelligence in Medicine), Biomedical Sciences and Imaging, Cedars-Sinai Medical Center, Los Angeles, CA USA; 3 Division of Cardiology, Department of Medicine, Department of Physiology, California NanoSystems Institute, David Geffen School of Medicine, University of California, Los Angeles (UCLA), Los Angeles, CA USA

**Keywords:** ^18^F-Flurpiridaz, Pharmacologic stress, Exercise stress, Positron Emission Tomography (PET), Myocardial Blood Flow (MBF), Quantitation, Myocardial Flow Reserve (MFR), Coronary Artery Disease (CAD)

## Abstract

**Purpose of Review:**

Recently approved by the United States Food and Drug Administration, ^18^F-flurpiridaz for positron emission tomography (PET) myocardial perfusion imaging (MPI) enables high-quality assessment of myocardial blood flow (MBF). Its half-life of approximately 110 min supports regional distribution and allows unit-dose delivery. These features make this probe well suited for MBF quantitation and expand access to quantitative cardiac PET at smaller centers.

**Recent Findings:**

During pharmacologic stress, absolute MBF and myocardial flow reserve provide incremental diagnostic information beyond relative perfusion imaging. These measures have been validated across multiple methods and software platforms. Further study of this tracer under peak exercise may provide clinically relevant assessment of myocardial demand-induced changes in flow. Potential investigational approaches include artificial intelligence (AI)-derived virtual MBF, exercise-adjusted standardized uptake value (SUV) ratio-derived myocardial flow reserve (MFR) surrogate, and direct kinetic modeling during in-scanner ergometry.

**Summary:**

While conceptually promising, limitations inherent to exercise-based approaches in stress MBF and/or MFR estimation highlight the need for pragmatic research strategies before potential clinical applicability.

## Introduction

**Fig. 1:**
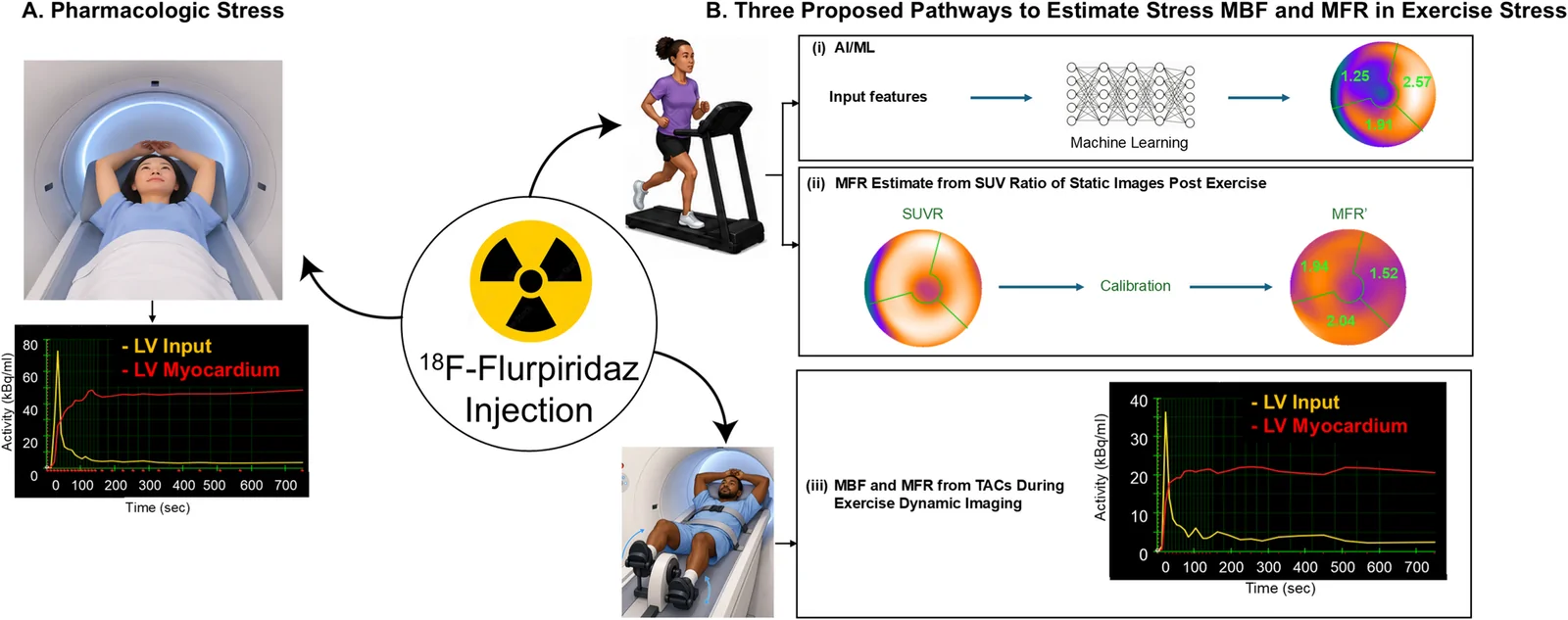
^18^F-Flurpiridaz PET strategies for quantifying or estimating myocardial blood flow and myocardial flow reserve. **A** Pharmacologic stress with dynamic PET acquisition. **B** Exercise stress: (i) virtual MBF/MFR estimated by artificial intelligence or machine learning from clinical data, static PET, and attenuation CT; (ii) MFR surrogate derived from post-exercise static images using stress-to-rest SUV ratio calibrated to MFR and factoring differences in ^18^F-Flurpiridaz myocardial SUVs between pharmacologic and exercise stress; (iii) in-scanner exercise with dynamic acquisition and novel kinetic modeling. Legend. AI: Artificial Intelligence, CT: Computed Tomography, LV: Left Ventricular, MBF: Myocardial Blood Flow, MFR: Myocardial Flow Reserve, MFR’: Myocardial Flow Reserve Estimate, ML: machine learning, PET: Positron Emission Tomography, SUV: standardized uptake value, SUVR: SUV ratio. TAC: time-activity curve

^18^F-flurpiridaz is the most recently approved positron emission tomography (PET) myocardial perfusion imaging (MPI) probe by the United States Food and Drug Administration (FDA) for clinical use in adults with known or suspected coronary artery disease (CAD) [1–7]. Beyond its indicated approval for relative perfusion imaging, properties of ^18^F-flurpiridaz render this probe highly suitable for myocardial blood flow (MBF) quantitation during pharmacologic stress. The tracer’s high myocardial extraction supports quantitative flow assessment [3, 6, 8, 9], which complements relative perfusion imaging by improving detection of multivessel CAD, assessment of coronary microvascular function, and risk stratification [10, 11]. Regional cyclotron production and unit-dose distribution are anticipated to broaden access to PET MPI [3], while established pharmacologic stress protocols facilitate first-pass MBF quantitation (Fig. 1A). Binding to mitochondrial complex I [12, 13] supports rapid myocardial uptake and prolonged retention, with 94% first-pass extraction reported in preclinical studies [14] and a near-linear relationship between uptake and MBF across a broad flow range [3, 8]. Its 110-min half-life permits injection at peak exercise followed by post-exercise imaging [9]. This review examines three emerging strategies that may enable exercise-stress MBF and/or MFR assessment with ^18^F-flurpiridaz (Fig. 1B): i) Artificial intelligence (AI)-derived virtual MBF prediction, ii) Standardized Uptake Value Ratio (SUVR)-based exercise MFR estimation, and iii) direct kinetic modeling during in-scanner exercise. We discuss the potential of each approach and the challenges to clinical implementation.

## Technical Considerations for Dynamic ^18^F-Flurpiridaz PET Imaging

^18^F has a positron range of 1.03 mm in water, shorter than ^82^Rb (8.6 mm), ^15^O (4.14 mm), and ^13^N (2.53 mm), resulting in improved spatial resolution of perfusion images [3, 9]. This may also increase the impact of motion on ^18^F-flurpiridaz quantitative MBF assessment, if not adequately corrected. Accordingly, motion correction, such as frame-by-frame methods or built-in correction during reconstruction, is necessary to ensure correct MBF estimates and CAD detection accuracy [15]. Automated dynamic motion correction was shown to improve the diagnostic performance of ^18^F-flurpiridaz PET MBF quantitation [16].

Recently, deep learning-based automated motion correction demonstrated diagnostic performance comparable to or better than expert manual correction, while reducing variability and processing time [17]. Importantly, given the long half-life of ^18^F, residual activity correction from the rest ^18^F-flurpiridaz injection needs to be conducted prior to the subsequent stress study. Failure to correct this rest residual activity may lead to overestimation of stress MBF and MFR, thereby adversely impacting diagnostic performance [14, 15, 17].

## Vasodilator Stress for Absolute ^18^F-Flurpiridaz MBF Quantitation in Humans

Following an initial human feasibility study of ^18^F-flurpiridaz MBF quantitation [6], three quantitative methods were developed and implemented in nuclear cardiology software [7, 15, 18]. Comparison of these methods [19] using data from the second phase 3 clinical trial [20] demonstrated substantial agreement and similar diagnostic performance [19]. No validated ^18^F-flurpiridaz-specific threshold distinguishes normal from abnormal resting MBF. For identifying ≥ 70% epicardial stenosis, hyperemic MBF below 1.6 mL/min/g with ECTb (Syntermed) or QPET (Cedars-Sinai), or below 1.7 mL/min/g with 4DM (Invia), should raise suspicion for CAD [19]. Corresponding MFR thresholds are 2.2 with ECTb or QPET and 2.1 with 4DM [19]. These thresholds have not been prospectively validated and at present should serve as diagnostic aids, not universal limits of normality.

A total-body PET study in healthy volunteers further characterized ^18^F-flurpiridaz kinetics at rest [21]. The investigators observed that a reversible 2T4K model, i.e. a two-tissue, four-rate-constant model, generally best captured multiorgan behavior. As previously described [19], the one-tissue compartment model provides a simplified framework for MBF assessment by representing myocardial tissue kinetics with a single exchangeable compartment and two rate constants: K1, describing tracer influx from the coronary circulation into myocardial tissue, and k2, describing tracer efflux or washout from tissue back to blood. In contrast, the two-tissue compartment model includes an additional, more slowly exchanging compartment representing retained or target-associated tracer. This model is parametered by four rate constants: K1 and k2, which characterize bidirectional exchange between plasma and the first tissue compartment, and k3 and k4, which describe transfer into and out of the second compartment, respectively, presumably reflecting mitochondrial complex I-associated binding or retention and its (negligible) reversibility for ^18^F-flurpiridaz. These findings demonstrate the potential of total body synchronous multi-organ assessment, supporting emerging cardiovascular PET/CT applications that extend beyond MPI [22].

### Clinical Integration of Pharmacologic Stress ^18^F-flurpiridaz MBF Measures

Absolute MBF quantitation with ^18^F-flurpiridaz during pharmacologic stress has demonstrated consistent diagnostic value across multicenter clinical studies. These investigations, predominantly based on angiographic reference standards (CAD ≥ 50% and ≥ 70% stenosis thresholds by quantitative invasive coronary angiography), demonstrate significant reductions in ^18^F-flurpiridaz MBF with increasing stenosis severity, and incremental value beyond relative perfusion imaging [6, 23]. However, reliance on % stenosis alone may limit alignment between PET MBF metrics and angiographic findings of disease [24, 25]. While segmental ^18^F-flurpiridaz flow analyses [7, 15, 17, 26] have demonstrated improved diagnostic performance over coronary territory measures [7], their utility in routine clinical practice remains to be established.

The multi-societal ^18^F-flurpiridaz procedure standard and practice guideline offers a practical framework for facilities implementing protocols for quantifying MBF during pharmacologic stress [9]. A 10-min rest acquisition followed by immediate pharmacological stress and a 10-min stress acquisition protocol was recently proposed, with early results indicating preserved diagnostic yield of relative perfusion and MBF quantitation [27, 28].

## Potential of ^18^F-Flurpiridaz MBF and MFR Estimation at Peak Exercise

Physiologic exercise stress provides incremental hemodynamic and electrocardiographic (ECG) information reflecting demand-induced ischemia, rather than vasodilator capacity. Accordingly, exercise stress is generally preferred over pharmacologic vasodilation in patients who can achieve an adequate cardiac workload. To date, however, exercise stress has precluded quantitative MBF assessment with ^18^F-flurpiridaz PET, because early tracer kinetics cannot be captured when the patient is outside the scanner. This limitation removes a major value-add of PET MPI. The 110-min half-life of ^18^F-flurpiridaz makes exercise PET MPI logistically feasible, as tracer administered at peak exercise retains sufficient activity for imaging after the patient returns to the scanner. Several approaches to estimate exercise PET MBF and/or MFR are currently under active investigation or may be considered, as illustrated in Fig. 1 and Table 1 and developed below.

**Table 1:** Proposed Exercise Stress Derived Myocardial Blood Flow Estimation Pathways for ^18^F-flurpiridaz PET

Approach	Input or Acquisition	Analytic Pathway	Quantitative Output
AI/ML	Clinical features; static PET; attenuation correction CT	Integrate the input features using machine learning	Virtual MBF; virtual MFR
Static-image SUVR calibration	Static PET images at rest and after exercise; myocardial SUVs	Calculate SUVR; establish the relationship between SUVR and true MFR under pharmacologic stress; correct for the difference in myocardial tracer uptake between pharmacologic and exercise stress (ratio approximately 2.2); derive exercise MFR’ from SUVR	Estimated MFR (MFR’)
Dynamic TAC modeling	Dynamic PET during exercise; LV input and myocardial TACs	Develop new biomathematical and kinetic models to determine MBF from exercise TACs; establish exercise-specific normal limits and significance thresholds for MBF and MFR; calculate MFR as exercise MBF divided by rest MBF	Absolute MBF at peak exercise; MFR

Abbreviations: *AI* artificial intelligence; *CT* computed tomography; *LV* left ventricular; *MBF* myocardial blood flow; *MFR* myocardial flow reserve; *ML* machine learning; *PET* positron emission tomography; *SUV* standardized uptake value; *SUVR* standardized uptake value ratio; *TAC* time-activity curve

### AI Developments

A strategy to address limitations in post-exercise imaging analysis involves machine learning (ML) or artificial intelligence (AI) to estimate virtual MBF and MFR from data extracted from routinely acquired static PET perfusion images, attenuation CT images, and clinical parameters, as has been developed for (pharmacologic stress) ^82^Rb-chloride PET MPI (Fig. 1Bi). The ML model has been evaluated on ^82^Rb-chloride dynamic PET data [29]. This AI-derived approach shows promising early results that may obviate the need for dynamic imaging. This could prove especially useful for exercise PET protocols where dynamic scans are logistically challenging or when the quality of time-activity curves is insufficient due to patient movement or technical issues.

To enhance clinical interpretation of MBF, a paired pilot study also compared AI-enabled MBF parametric mapping using ^82^Rb-chloride PET with ^18^F-flurpiridaz PET, supporting cross-tracer translation of AI-based flow approaches [30]. This method may be ideally suited for exercise PET protocols using ^18^F-flurpiridaz to provide improved visualization of MBF beyond standard polar map representation, however, has not been evaluated to date.

AI offers a promising opportunity to further enhance the diagnostic accuracy of ^18^F-flurpiridaz PET. Although sufficiently large datasets are not yet available to develop robust multimodal AI models for ^18^F-flurpiridaz, experience with ^82^Rb-chloride PET provides a strong proof of concept. AI models trained using the large multicenter REFINE PET registry [31] have shown that integrating complementary PET biomarkers – including myocardial perfusion, myocardial blood flow, myocardial flow reserve, ventricular function, and coronary artery calcium (CAC) improves diagnostic accuracy compared with individual imaging parameters alone [32]. As larger ^18^F-flurpiridaz datasets become available, similar AI approaches could be developed to integrate these quantitative imaging biomarkers together with clinical variables and the unique advantages of exercise PET. Supporting this concept, a recent study demonstrated that combining AI-derived CAC with ^18^F-flurpiridaz MPI improved diagnostic accuracy [33]. However, AI models integrating myocardial blood flow and myocardial flow reserve with perfusion and other imaging biomarkers have not yet been developed for ^18^F-flurpiridaz. Such multimodal AI approaches may further improve the detection of obstructive coronary artery disease.

### Standardized Uptake Values (SUV) Ratio-Derived Estimated MFR

Conventional kinetic modeling requires capturing tracer first-pass kinetics at the time of injection. However, once post-exercise imaging is performed, the tracer has already spread into the myocardium. This makes it impossible to recover the arterial input function and invalidates MBF calculations based on this method, requiring an alternative approach. Using an ^18^F-flurpiridaz SUV-ratio-based MFR surrogate has been proposed [34].

SUV is a semi-quantitative PET measure of tracer concentration normalized to injected dose and body size according to the following formula:

$$SUV=\frac r{\left({a'}\diagup w\right)}$$

where *r* is the tissue radioactivity concentration [kBq/mL], which is measured by the PET scanner within a region of interest (ROI); *a*′ denotes the decay-corrected injected dose [kBq]; and *w* indicates the weight of the patient [g], used as a surrogate for the volume of the radiotracer distribution. By normalizing radiotracer uptake, SUV provides a standardized and reproducible measure that facilitates comparisons across serial scans and also different patients. The commonly used forms of SUV include SUV_mean_, SUV_max_, and SUV_peak_, which differ in how activity is averaged within the myocardium. Although relatively simple and widely used, SUV does not directly measure MBF and is influenced by numerous technical and physiologic factors including reconstruction parameters, motion, scanner calibration, uptake time, and patient body composition and physiology, limiting its specificity.

The stress-to-rest ratio of SUV (SUVR) is proportional to MFR and was proposed as a ‘rough’ proxy for MFR (Fig. 1Bii). This concept was initially tested in pig models [35]. The study showed that SUVs measured 5 to 12 min after ^18^F-flurpiridaz injection correlated strongly with reference MBF obtained via microspheres (*r* = 0.92, *p* < 0.001). Additionally, the surrogate MFR estimated from rest and stress SUVs closely matched microsphere-derived MFR. An early feasibility study evaluated MFR estimation from exercise stress ^18^F-flurpiridaz perfusion data in a cohort of 220 patients who underwent either pharmacologic or treadmill stress testing [34]. The investigators modeled the relationship between stress-to-rest SUVR and ‘true’ MFR in pharmacologic stress studies using a power function with cross-validation. This was followed by correction for differences in myocardial SUVs between ^18^F-flurpiridaz pharmacologic stress and ^18^F-flurpiridaz exercise stress [36]. Initial results suggest improved detection of multivessel CAD in exercise stress ^18^F-flurpiridaz using this MFR estimation. Indeed, when SUV is used to derive SUVR, differences between rest and exercise conditions can introduce bias. In addition, SUVR needs to be adjusted to account for physiological differences between exercise and pharmacologic stress. Variations in treadmill protocols, injection timing, post-exercise intervals, and individual responses call for standardized reference ranges. There is also a lack of standardized quality control frameworks for exercise-stress MBF estimation. SUVR-based estimates of MFR remain indirect and require calibration against validated flow measurements. Evaluation of this technique will be warranted across diverse cohorts.

### Possibility of In-Scanner Exercise Stress with Dynamic Acquisition

Research on quantitative PET MBF assessment during exercise remains limited, with available data mostly derived from small feasibility studies using in-scanner supine bicycle ergometry. Early work with ^13^N-ammonia demonstrated the feasibility of dynamic PET acquisition during exercise, showing an approximately two-fold increase in MBF in healthy volunteers [37]. Subsequent studies with ^15^O-water and ^82^Rb-chloride confirmed that exercise MBF measurements are reproducible and technically feasible [38, 39]. However, exercise-induced hyperemia was substantially lower than that achieved with adenosine, reflecting demand-mediated rather than pharmacologic vasodilatory flow augmentation. The repeatability coefficient, reflecting expected variability between repeated measurements, was lower for exercise stress MBF than for adenosine stress MBF, suggesting consistent exercise-derived flow measurements across repeated scans [38].

Despite these proof-of-concept studies, in-scanner exercise PET MBF quantitation has not been adopted, largely because of technical challenges related to patient motion, the need for sustained exercise within the scanner, novel kinetic modeling, tracer-specific logistical constraints, and the now large body of evidence supporting vasodilator stress MBF quantitation. Measuring MBF during peak exercise requires tracer injection during active stress and dynamic image acquisition while the patient exercises within the PET scanner, typically using a supine bicycle ergometer compatible with the scanner bore (Fig. 1Biii). Implementation of this approach in clinical practice would require specialized ergometers. It will also need ^18^F-flurpiridaz injection timing during peak workload to ensure reliable data acquisition. Future studies should evaluate such in-scanner exercise stress ^18^F-flurpiridaz PET kinetic modeling and MBF quantitation.

### Considerations for Clinical Translation

Several methodological and technical constraints temper the clinical implementation of exercise-based MBF estimation. Novel kinetic modeling and/or novel software approaches such as virtual MBF will be required for exercise stress MBF modeling. Additional limitations arise from reliance on semi-quantitative metrics such as SUV, which do not directly measure MBF and remain sensitive to acquisition conditions, as described above. When these metrics are extended to stress-to-rest ratios, the problem may become greater, since rest and exercise differ physiologically and exercise stress protocols are not as standardized as pharmacologic stress protocols.

## Conclusion

^18^F-flurpiridaz is a novel US FDA-cleared PET myocardial perfusion agent with favorable properties for quantitative MBF assessment. While pharmacologic stress MBF quantitation with this radiopharmaceutical is already supported by validated approaches and used clinically, its 110-min half-life opens an additional and potentially transformative opportunity for exercise-stress PET with assessment of demand-induced flow responses. This capability may extend the value of PET MPI beyond conventional vasodilator physiology and enable a more complete interrogation of ischemia under true physiologic stress. Of note, while not developed in this review, combining a standard exercise stress ECG, i.e. with no imaging component, with vasodilator stress ^18^F-flurpiridaz PET/CT, while not ideal, remains feasible.

Each approach offers a distinct path forward and will require rigorous technical validation, clinical evaluation, and standardization before potential clinical implementation. Together, however, they point toward an exciting next phase in quantitative cardiac PET imaging. By combining broad accessibility, high image quality, validated pharmacologic stress MBF quantitation, and the potential for exercise-based flow assessment, ^18^F-flurpiridaz is poised to help expand patient access to quantitative PET MPI and advance the physiologic assessment of ischemic heart disease.

## Key References

- Packard RRS. The clinical promise of ^18^F-flurpiridaz PET imaging heralds a new frontier in the diagnosis and management of coronary artery disease. Nat Cardiovasc Res. 2025;4:1–4. https://doi.org/10.1038/s44161-024-00587-w.
  - ○ This paper summarizes the key attributes of ^18^F-flurpiridaz for pharmacologic and exercise stress PET imaging.
- Urs M, Kwiecinski J, Lemley M, Chareonthaitawee P, Ramirez G, Shanbhag A, et al. Virtual myocardial blood flow and flow reserve from static PET imaging using artificial intelligence. medRxiv. 2026. https://doi.org/10.64898/2026.02.03.26345376.
  - ○ This work indicates the feasibility of using artificial intelligence to estimate virtual myocardial blood flow from static images in ^82^Rb-chloride PET.

## Data Availability

No datasets were generated or analysed during the current study.
